# L’hyperthyroïdie de l’enfant au centre hospitalier universitaire de Dakar (Sénégal)

**DOI:** 10.11604/pamj.2017.28.10.13396

**Published:** 2017-09-07

**Authors:** Djibril Boiro, Demba Diédhiou, Babacar Niang, Djiby Sow, Mandiaye Mbodj, Anna Sarr, Aliou Abdoulaye Ndongo, Aliou Thiongane, Modou Guèye, Lamine Thiam, Ndiogou Seck, Yaay Joor Dieng, Abou Ba, Idrissa Demba Ba, Ibrahima Mané Diallo, Ousmane Ndiaye, Said Nourou Diop

**Affiliations:** 1 Service de Pédiatrie et Néonatologie Centre Hospitalier Abass Ndao/UCAD, Dakar, Sénégal; 2 Clinique Médicale II/Centre Hospitalier Abass Ndao/UCAD, Dakar, Sénégal; 3 Centre Hospitalier National d’Enfants Albert Royer/UCAD, Dakar, Sénégal; 4 Centre Hospitalier de la Paix/UFR Santé, Ziguinchor, Sénégal; 5 Centre Hospitalier Régional de Saint Louis/UFR santé, Dakar, Dakar

**Keywords:** Hyperthyroïdie, enfant, Sénégal, Hyperthyroidism, child, profile, Senegal

## Abstract

**Introduction:**

L'hyperthyroïdie de l'enfant semble rare et constitue un problème de par son retentissement psychosomatique. L’objectif était de faire le point sur ses aspects épidémiologiques et diagnostiques chez l’enfant à Dakar.

**Méthodes:**

Il s'agissait d'une étude multicentrique, descriptive sur 15 ans. Etaient analysés les aspects épidémiologiques, cliniques et étiologiques.

**Résultats:**

239 patients sélectionnés avec une prévalence de 2.4%, un sex ratio (H/F) de 0.36, un âge moyen de 10.8 ans. À l'inclusion, il s'agissait d'un ainé de famille (26.3%), d'une croissance avancée (36.9%), retardée (12.5%), d'une corpulence insuffisante (40.1%). L'étiologie était la maladie de Basedow dans 90.3% avec un facteur psychoaffectif dans 22.1%. Sur le plan clinique, prédominaient la tachycardie (92.4%), le goitre (91.1%), l'exophtalmie (81.8%), l'amaigrissement (69.8%) avec cependant une énurésie (30.2%) et des manifestations psychiques (3.1%). Les manifestations cardiovasculaires et cutanées étaient positivement associées avec l'âge (p < 0.05). Le goitre était associé au sexe féminin (p = 0.005), aux signes cardiovasculaires (p = 0.02), neuropsychiques (p = 0.03), cutanées (p = 0.03) et à la diarrhée (p = 0.03). La T4 libre était corrélée à l'âge (p = 0.007), la diarrhée (p = 0.021), l'anxiété (p = 0.024), la fréquence cardiaque (p = 0.00) et la maladie de Basedow (p = 0.04). Plus le goitre était volumineux, plus était augmentée la T4 libre (p = 0.007).

**Conclusion:**

Conclusion: L'hyperthyroïdie de l'enfant se différencie de celle de l'adulte par les facteurs d'induction, les perturbations sur la croissance et l'énurésie. L’âge et le sexe semble favoriser le tableau clinique de thyrotoxicose et les signes associés.

## Abstract (en)

**Introduction:**

Hyperthyroidism in children is rare and constitutes a problem because of its psychosomatic impact. This study aimed to present an overview of the state of knowledge on these aspects in children from Dakar.

**Methods:**

We conducted a multicenter, descriptive study over a period of 15 years. We evaluated the epidemiological, clinical and etiologic aspects of hyperthyroidism in children

**Results:**

239 patients were included in the study with a prevalence of 2.4%, a sex ratio (M/F) of 0.36 and an average age of 10.8 years. The inclusion criteria were being the eldest son in a family(26.3%), being advanced (36.9%) or delayed (12.5%) in maturation, having short stature (40.1%). It was caused by Graves' disease in 90.3% of cases with psychoaffective factor in 22.1%. On a clinical point of view tachycardia (92.4%), goiter (91.1%), exophthalmia (81.8%), weight loss (69.8%) prevailed but with bed-wetting (30.2%) and psychic manifestations (3.1%). Cardiovascular and cutaneous manifestations were positively associated with age (p < 0.05). Goiter was associated with female sex (p = 0.005), cardiovascular (p = 0.02), neuropsychic (p = 0.03), skin (p = 0.03) signs and diarrhea (p = 0.03). Free T4 was correlated with age (p = 0.007), diarrhea (p = 0.021), anxiety (p = 0.024), heart rate (p = 0.00) and Graves' disease (p = 0.04). More voluminous the goiter, higher the free T4 (p = 0.007).

**Conclusion:**

Hyperthyroidism in children differs from that in adults because of induction factors, growth disturbances and enuresis. Age and sex seem to favor the clinical picture of thyrotoxicosis and its associated signs.

## Introduction

L'hyperthyroïdie atteint 1 à 2% de la population mondiale, mais semble rare et sévère chez l'enfant [1]. Elle est due le plus souvent à la maladie de Basedow, maladie auto-immune qui résulte de la stimulation des récepteurs thyréotropes par des auto-anticorps [2]. L'hyperthyroïdie peut être néonatale avec une expression clinique et physiopathologique particulière ou parfois atteindre l'enfant plus âgé. Sur ce terrain, elle constitue un problème de santé publique à cause de son retentissement psychosomatique et ses répercussions sur la croissance et la puberté [3,4]. Compte tenu du nombre croissant de patients présentant cette affection dans notre pratique, nous avons jugé opportun, de faire le point en se fixant comme objectif, l'évaluation des aspects épidémiologiques, cliniques et étiologiques dans les services de références en endocrinologie au Centre Hospitalier et Universitaire de Dakar.

## Méthodes

Il s'agissait d'une étude multicentrique, transversale, descriptive et analytique réalisée au Centre Hospitalier National d'Enfants Albert Royer, à la Clinique Médicale II du Centre Hospitalier Abass Ndao et au service de pédiatrie du Centre Hospitalier Abass Ndao de Dakar. Elle concernait les patients âgés de 15 ans au plus suivis du 1er janvier 2000 au 31 décembre 2014 (15 ans). Etaient inclus, les enfants porteurs d'une hyperthyroïdie confirmée sur le plan biologique et suivis dans l'un des différents services. Les dossiers incomplets étaient exclus de l'analyse finale. Le diagnostic positif de l'hyperthyroïdie était retenu sur l'existence d'un tableau clinique de thyrotoxicose associé à une augmentation des hormones thyroïdiennes (tétra-iodothyronine (T4) libre et/ou la triiodothyronie (T3) libre). La forme topographique était retenue sur le taux de thyréostimuline (TSHus). La maladie de Basedow correspondait à l'association au syndrome de thyrotoxicose, d'un goitre diffus, vasculaire, d'une exophtalmie acquise, d'un myx'dème prétibial. Le dosage des anticorps anti récepteurs de la thyréostimuline n'était pas effectué chez tous les patients. L'adénome toxique était retenu si à l'hyperthyroïdie périphérique s'associait un nodule thyroïdien unique. Un goitre multinodulaire toxique était l'étiologie si à l'hyperthyroïdie périphérique s'associait un goitre multinodulaire à l'échographie.

Les paramètres étudiés étaient les suivants: les aspects épidémiologiques: l'âge, le sexe, la fratrie, la scolarité, la région de provenance, les facteurs psychoaffectifs. Pour ces derniers, il s'agissait de l'étude de facteurs déclenchant ou d'auto entretien de l'hyperthyroïdie surtout dans le cadre de la maladie de Basedow. Nous avons regroupé les patients en trois catégories: catégorie 1 (conflits socio-familiaux: divorce parental, difficultés au sein de la famille), catégories 2 (difficultés scolaires), catégorie 3 (chocs psychoaffectifs: décès ou maladie d'un proche, éloignement des parents ou confiage, sevrage); Les éléments cliniques concernaient les données anthropométriques, les signes de thyrotoxicose et ceux propres aux étiologies (caractéristiques du goitre selon la classification de l'Organisation Mondiale de la Santé [5], de l'ophtalmopathie, de la dermopathie prétibiale). Nous avons aussi évalué le délai de consultation, les manifestations cliniques au début et leur évolution pendant le suivi; Les données paracliniques étaient relatives à la valeur de la T3 libre, la T4 libre, la TSH ultra-sensible au début et leur évolution pendant le suivi. Les valeurs de la TSH et des hormones thyroïdiennes étaient déterminées à jeun par les méthodes immuno-radiologiques. Les normes biochimiques de notre laboratoire étaient de 0.17 à 4.05 mUI/l pour la TSH, de 9 à 22 pmol/l pour L-T4 et de 2.5 à 5.8 pmol/l pour la L-T3. Les résultats de l'imagerie (échographie cervicale) étaient aussi pris en compte; Pour l'analyse descriptive les données étaient présentées en pourcentage pour les variables qualitatives et en moyennes avec Ecart-type pour les variables quantitatives. Les tests statistiques usuels utilisés étaient le Test de chi-2 pour les variables qualitatives et celui de Student pour les variables quantitatives. Un p < 0.05 était considéré comme statistiquement significatif avec un intervalle de confiance (IC) à 95%; Ce travail a reçu l'approbation du Comité National d'Ethique pour la Recherche en Santé (CNERS) du Sénégal et conformément aux procédures établies par l'Université Cheikh Anta DIOP de Dakar (UCAD).

## Résultats

**Aspects épidémiologiques:** Durant l'étude, nous avions colligé 239 patients dont 87 (36.4%) au Centre Hospitalier National d'enfant Albert Royer et 152 (63.6%) à l'hôpital Abass Ndao. La prévalence de l'hyperthyroïdie chez l'enfant était de 2.4% parmi les 9700 patients tous âges confondus suivis pour une thyréopathie. La prédominance était féminine avec un sex ratio (H/F) de 0.36. L'âge moyen était de 10.8 ans (extrêmes de 0.6 ans et 15 ans). L'âge était compris entre 0 et 5 ans dans 30 cas (12.5%), de 6 à 11 ans dans 79 cas (33%) et de 12 à 15 ans dans 130 cas (54.4%). Deux cas (0.8%) d'hyperthyroïdies néonatales basedowiennes étaient retrouvés. Les patients étaient des élèves dans 195 cas (81.5%). Un abandon scolaire était retrouvé dans 14 cas (5.8%). Trente-deux enfants (dont 93.7% avaient moins de 6 ans) n'étaient pas scolarisés. Ils résidaient dans la région de Dakar dans 165 cas (69%). Concernant le rang dans la fratrie, il s'agissait d'un ainé (26.3%), d'un deuxième enfant (21.8%), d'un troisième enfant (18.8%), d'un troisième ou quatrième enfant (21%). Les deux parents étaient tous vivants dans 234 cas (97.9%).

**Aspects étiologiques:** Une dysthyroïdie familiale était retrouvée dans 43 cas (18%). Un facteur psychoaffectif était retrouvé dans 53 cas (22.1%). Il concernait 22.2% des filles, 21.8% des garçons, les enfants de 10 ans et plus dans 73.5% des cas. Parmi ces 53 enfants chez qui était retrouvé un facteur d'induction, la catégorie 3 représentait 60.3% des cas. La catégorie 1 était retrouvée dans 35.8% des cas. Dans la majorité des cas (82%), l'interrogatoire n'a pas permis de retrouver un facteur favorisant. L'étiologie de l'hyperthyroïdie était la maladie de Basedow (90.4%), l'adénome toxique (2.5%) et le goitre multinodulaire toxique (7.1%) dont onze cas de goitre multinodulaire basedowifiés (4.6%) (Tableau 1).

**Tableau 1:** Répartition selon l’étiologie de l’hyperthyroïdie

Etiologies	Effectif (n=239)	Pourcentage (%)
Maladie de basedow	216	90.4
Adénome toxique	6	2.5
Goître multinodulaire	17	7.1

**Aspects cliniques à l'inclusion:** Le délai moyen de consultation dans nos services respectifs était de 12.5 mois. Ce délai était moins de 6 mois dans 87 cas (36.4%), de 6 à 10 mois dans 100 cas (41.8%), de 13 à 24 mois dans 27 cas (11.2%) et de plus de deux ans dans 25 cas (10.4%). Concernant les données anthropométriques, la croissance des enfants à l'inclusion était normale 50.6%, avancée dans 36.9% et retardée dans 12.5% des cas. Selon l'index de masse corporelle, la corpulence était normale dans 53.1%, insuffisante (insuffisance pondérale) dans 40.1%. Le surpoids concernait 6.8% des patients. Sur le plan de la thyrotoxicose, la tachycardie était quasi constante (93.3%). Les autres signes cliniques les plus fréquents étaient l'amaigrissement (69.8%), l'asthénie (62.2%), les tremblements (58.7%), les palpitations (54.7%), la dyspnée d'effort (54.7%), l'hypersudation (41.8%), la thermophobie (36.9%), la nervosité et l'irritabilité (38.2%) et la diarrhée (24.4%). Une énurésie était retrouvée dans 30.2%, des troubles du sommeil dans 20.0% et des manifestations psychiques dans 3.1% (Tableau 2). Les signes cliniques étaient plus marqués chez ceux qui avaient consultés dans les dix premiers mois sans corrélation significative (p > 0.05). Les manifestations cliniques comme les palpitations, la dyspnée, la thermophobie, l'hypersudation et le tremblement étaient significativement associées avec l'âge. Plus celui-ci augmentait, plus ces signes devenaient importants (p < 0.05). Les autres signes cliniques associés à la thyrotoxicose étaient un goitre dans 91.1%. Ce goitre était de grade 2 (71.1%), de grade 3 (12.9%), bilatéral (62.2%), diffus (86.2%), vasculaire (58.2%), nodulaire (3.1%), multinodulaire (0.4%), douloureux (1.3%) et compressif (1.2%). Dix-neuf patients (7.9%) avaient une thyrotoxicose sans goitre. Le goitre était significativement associé au sexe féminin (p = 0.005), à la polyurie (p = 0.02), aux signes cardiovasculaires (p = 0.02), neuropsychiques (p = 0.03), cutanéo-phanériennes (p = 0.03) et à la diarrhée (p = 0.03). Le grade du goitre était significativement associé à tous les signes cliniques. Plus les signes cliniques étaient intenses, plus le goitre était volumineux (p < 0.05). L'exophtalmie était présente chez 81.8% des patients. Elle était bilatérale (70.7%), réductible (80%) et non inflammatoire (99.6%). Les paramètres associés à l'exophtalmie étaient la dyspnée d'effort (p = 0.027), les caractères vasculaire (p = 0.005), diffus (p = 0.000) du goitre. La dermopathie prétibiale concernait 2 patients (0.9%).

**Tableau 2:** Répartition des signes cliniques à l’inclusion

Signes Cliniques	Effectif (n=239)	Pourcentage (%)
Tachycardie	223	93.3%
Palpitation	137	57.3%
Dyspnée	125	52.3%
Précordialgies	6	2.5%
Amaigrissement	170	71.1%
Asthénie	149	62.3%
Polyphagie	53	22.1%
Polyurie	46	19.2%
Polydipsie	40	16.7%
Signe du Tabouret	36	15.0%
Tremblement	146	61.0%
Nervosité	98	41.0%
Irritabilité	97	40.5%
Insomnie	58	24.2%
Anxiété	50	20.9%
Troubles psychiatriques	7	2.9%
Hypersudation	107	44.7%
Thermophobie	96	40.1%
Mélanodermie	35	14.6%
Enurésie	75	31.3%
Diarrhée	61	25.5%
Dysménorrhée	13	5.4%
Goitre	220	92.0%
Exophtalmie	195	81.5%
Dermopathie prétibiale	2	0.8%

**Aspects paracliniques à l'inclusion:** La confirmation de l'hyperthyroïdie à l'inclusion s'était aidée de la TSHus (96.9%), la T4 libre (93.3%), la T3 libre (36.9%). Toutes les valeurs de TSHus étaient inférieures à 0.02 mUI/l. Parmi les patients ayant bénéficié d'un dosage de la T4 libre au diagnostic, cette dernière était entre 23 et 40 pmol/l (20.9%), entre 41 et 60 pmol/l (27.1%), entre 61 et 80 pmol/l (25.2%), entre 81 et 100 pmol/l (20.4%) et au-delà de 100 pmol/l (6.2%). Les manifestations de thyrotoxicoses les plus significativement corrélée à la valeur de la T4 libre au diagnostic était la diarrhée (p = 0.021), l'anxiété (p = 0.024) et la fréquence cardiaque (p = 0.00). La maladie de Basedow était l'étiologie la plus positivement corrélée à la valeur de la T4 libre (p = 0.04). Dans ce cadre précis de maladie de Basedow, plus le goitre était volumineux, plus était augmentée la valeur de la T4 libre (p = 0.007). La valeur de la T4 libre augmentait aussi avec l'âge des enfants (p = 0.044) et la présence d'une exophtalmie. L'échographie cervicale réalisée chez 220 patients (92%) retrouvait un goitre diffus (96.3%), vasculaire (87.2), uni nodulaire (2.7%), multinodulaire (7.7%).

## Discussion

Dans notre série, la prévalence de l'hyperthyroïdie était de 2.4% parmi toutes les thyréopathies. En Afrique Sub Saharienne, une série de 38 cas colligés au Mali, rapportait une fréquence à 0.96% parmi les hyperthyroïdies [6]. Ailleurs, des fréquences de 0.3% au Danemark [7], 1.2% aux Etats-Unis [8], 2.6% en Suède [9] ont été rapportées parmi les hyperthyroïdies tous âges confondus. L'hyperthyroïdie chez l'enfant, quel qu'en soit la forme étiologique prédomine dans le sexe féminin [2, 7-9]. Cette donnée a été confirmée par notre étude dans laquelle le sexe féminin prédominait avec un sex ratio de 0.36%. En Afrique, le sex ratio était de 0.33; 0.4; 0.0^8^ respectivement au Mali [6] en Tunisie [10] et en Algérie [11]. La moyenne d'âge était de 10.8 ans dans notre série. L'hyperthyroïdie peut se voit à tout âge avec un pic à l'adolescence [12]. Dans la plupart des études sur l'hyperthyroïdie liée à la maladie de basedow chez l'enfant, il est rapporté une prédominance des âges supérieurs ou égaux à 10 ans [2, 11, 13]. C'est le cas de la série de Sidibé et coll. [6] au Mali, où les auteurs rapportaient une prédominance chez les adolescents dans 65.7% des cas. La maladie de Basedow est plus fréquente chez les enfants porteurs de conditions auto immune et d'antécédents familiaux de dysthyroïdie auto immune [12, 14]. Dans notre série, une dysthyroïdie familiale était retrouvée dans 17.^9^% et concernait la maladie de Basedow dans 100% des cas. Boiko et coll [15], en avaient retrouvé dans plus de la moitié des cas de leur série. L'étiologie la plus fréquente des hyperthyroïdies demeure la maladie de Basedow quel que soit l'âge et le sexe. Elle était retrouvée dans 90.3% des cas de notre série. Ce résultat semble légèrement au-dessus des 84.2% rapportés par Sidibé et coll [6] au Mali, mais concordent cependant avec la plupart des données de la littérature africaine et européens [1, 2, 10, 11]. Le délai moyen de consultation à 12.5 mois dans notre étude concordait avec les données de Sy-Akossou et coll. [16] au Togo. Ailleurs, Boiko et coll [15] rapportaient un délai moyen du diagnostic de 3.5 mois. Le retard constaté dans nos régions est généralement en rapport avec une méconnaissance de la maladie mais aussi le recours au traitement traditionnel et aux difficultés liées à l'accès aux soins.

Les manifestations cliniques les plus fréquemment observée dans notre série concordent avec les données de la Littérature [11, 17]. La particularité chez l'enfant réside dans les troubles anthropométriques, le retentissement scolaire [18] et surtout l'énurésie retrouvée dans 31% dans notre série. Il est rapporté une avance staturale avec dans la moitié des cas une avance de la maturation osseuse. L'évolution en parallèle de la taille et de la maturation osseuse empêche la survenue d'une grande taille finale. La soudure prématurée des cartilages de croissance va finalement donner une petite taille finale. Le périmètre crânien doit être particulièrement surveillé chez le nourrisson car il s'opère une craniosténose qui entraine, de plus, une diminution des possibilités intellectuelles. Dans notre étude, la croissance staturale était avancée dans 36.9% des cas. Meziani et coll [19], rapportaient une avance staturale dans ¾ des cas. Comme dans notre série, la fréquence du goitre reste supérieure à 90% [6]. L'exophtalmie notée chez 81.8% des patients, est loin supérieure des 58.9% rapportés par Bhadada et coll [3] en Inde. La maladie de Basedow était l'étiologie la plus positivement corrélée à la valeur de la T4 libre (p = 0.04). Compte tenu de l'accès limité aux dosages hormonaux dans nos régions, les dosages seuls de la T4 libre et de la TSHus semblent raisonnables pour poser le diagnostic. Ces deux marqueurs sont suffisants pour confirmer le diagnostic d'hyperthyroïdie dans plus 95% des cas. Le dosage de T3 totale n'étant nécessaire que dans les rares cas d'altérations de la TBG que le seul dosage des formes libres conduirait à méconnaître [20-22]. Chez l'enfant, la sensibilité et la spécificité étiologiques de l'échographie dans la maladie de Basedow [8] justifie dans nos régions à ressources limité un moindre recours aux autres explorations étiologiques telles que la scintigraphie, le dosage de anticorps anti récepteurs de la TSH.

## Conclusion

L'hyperthyroïdie constitue un problème majeur chez les enfants compte tenu de ses répercussions graves sur la croissance et le développement de ces derniers. Elle se différencie de celle de l'adulte par les facteurs d'induction. L'âge et le sexe semblent favoriser le tableau clinique de thyrotoxicose et les signes associés. Il est important d'améliorer les moyens diagnostiques aussi bien cliniques que paracliniques afin de pouvoir établir un diagnostic précoce et par conséquent améliorer le pronostic.

### Etat des connaissances actuelles sur le sujet

- Pathologie rare mais sévère chez l'enfant;
- Maladie de basedow est l'étiologie prédominante;
- Retentissements graves sur la croissance et le développement de l'enfant.

### Contribution de notre étude à la connaissance

- Permettra de faire le diagnostic de plus en plus précoce en tenant compte de certains signes révélateurs de la maladie: l'énurésie, les troubles de la croissance staturale, l'absence de goitre dans certaines thyrotoxicoses;
- Permettra d'améliorer le bien être le pronostic des patients.

## Conflits d’intérêts

Les auteurs ne déclarent aucun conflit d'intérêts.

## Remerciements

A tous ce qui ont contribué à l'élaboration de ce travail.
